# Implementation of patient discharge management in a university hospital through action research

**DOI:** 10.1590/0034-7167-2025-0174

**Published:** 2026-10-19

**Authors:** Lélia Mendes Sobrinho de Oliveira, Larissa Chaves Pedreira, Ana Paula Santos de Jesus, Elizabeth Bernardino, Cristina Rosa Soares Lavareda Baixinho, Valdenir Almeida da Silva, Amanda Aparecida Silva Rios, Emanuela Santos Oliveira

**Affiliations:** I Universidade Federal da Bahia. Salvador, Bahia, Brazil; II Universidade do Recôncavo Baiano. Cruz das Almas, Bahia, Brazil; III Universidade Federal do Paraná. Curitiba, Paraná, Brazil; IV Escola Superior de Enfermagem de Lisboa. Lisboa, Portugal

**Keywords:** Transitional Care, Safety Management, Health Services Research, University Hospital, Patient Discharge., Cuidado de Transición, Administración de la Seguridad, Investigación sobre Servicios de Salud, Hospitales Universitarios, Alta del Paciente.

## Abstract

**Objectives:**

to establish and implement a Hospital Discharge Management Committee in a university hospital, and to assess the group’s perception of the committee’s planning and implementation.

**Methods:**

a qualitative study, conducted through action research. It took place in a university hospital in northeastern Brazil, over 12 months, following four phases: situational diagnosis; action planning; action implementation; and assessment.

**Results:**

the committee was established and implemented in the hospital, with a collaboratively constructed work process. Instruments and workflows were developed, including eligibility criteria, patient recruitment and admission procedures, a readiness scale, as well as follow-up, discharge, and post-discharge workflows, structuring a model to support continuity of care.

**Final Considerations:**

the committee was implemented to monitor patients in the pilot unit. In the group’s perception, there was an improvement in patient and caregiver monitoring, as well as a positive assessment of committee formation and methodology used, valuing each individual contribution.

## RESUMEN

**Objetivos:**

establecer e implementar un Comité de Gestión de Altas Hospitalarias en un hospital universitario y evaluar la percepción del grupo sobre la planificación e implementación del comité.

**Métodos:**

estudio cualitativo, realizado mediante investigación-acción, en un hospital universitario del noreste de Brasil, durante 12 meses, siguiendo cuatro fases: diagnóstico situacional; planificación de acciones; implementación de acciones; y evaluación.

**Resultados:**

el comité se estableció e implementó en el hospital, con un proceso de trabajo colaborativo. Se desarrollaron instrumentos y flujos de trabajo, incluyendo criterios de elegibilidad, procedimientos de reclutamiento y admisión de pacientes, una escala de preparación, así como flujos de trabajo de seguimiento, alta y postalta, estructurando un modelo para apoyar la continuidad de la atención.

**Consideraciones Finales:**

el comité se implementó para el seguimiento de los pacientes en la unidad piloto. El grupo percibió una mejora en el seguimiento de pacientes y cuidadores, así como una evaluación positiva de la formación del comité y la metodología empleada, valorando cada contribución individual.

## INTRODUCTION

Hospital discharge management is an administrative tool used to promote effective interventions to integrate points in the Health Care Network (In Portuguese, *Rede de Atenção à Saúde* - RAS), ensuring continuity of care^(1)^. It involves planning and structuring the discharge process, as well as providing guidance to family members and caregivers, and identifying and coordinating with the RAS^(2)^. This discussion is particularly necessary in developed and developing countries due to the aging population and the increased functional dependence of this age group, especially after long periods of hospital stays.

The Brazilian National Hospital Care Policy^(3)^ emphasizes responsible hospital discharge through the transfer of care. Despite this, this debate is recent in the country. In clinical practice, few actions on the subject are observed, especially in the public network, where the population needs it most. Moreover, this gap is also reported by national authors^(4,5)^, requiring intensification, especially in the context of the vulnerable population, with low income and educational level, with little formal or social support.

The lack of adequate planning for hospital discharge of vulnerable individuals, which involves continuity of care, is linked to serious adverse effects such as medication errors, delays in care, duplication of treatment, reduced adherence, worsening quality of life for the person and their caregiver, avoidable readmissions, inappropriate use of services, and increased healthcare costs^(4)^. Therefore, managing hospital discharge facilitates continuity of care and the efficient use of hospital and community resources, based on identifying and addressing the needs of patients and their caregiver, considering their life contexts^(5)^.

International experiences with discharge management for continuity of care have been reported in countries such as Spain^(6)^, Portugal^(7)^, and Canada^(8)^. In Brazil, a study conducted with professionals from the multidisciplinary team to develop and validate a checklist aimed at organizing hospital discharge indicates positive results after coordinated work to develop multidisciplinary discharge plans according to the needs of patients and caregivers. The authors emphasize that the result can be more satisfactory when discharge plans are articulated between the different levels of health care^(2)^.

In this context, nurses stand out in continuity of care coordination and development, given their managerial skills and competencies, health education, as well as their articulation with other professionals on the team and greater time spent in contact with patients and families. Furthermore, they possess knowledge of the healthcare systems and services available for follow-up care, making it important to deepen the study of transitional care and their potential role as multidisciplinary team members^(1,4,8)^.

Therefore, there is an urgent need to implement strategies to ensure a safe transition between hospital and home, improving multidisciplinary discharge plans in work processes, as well as in the country’s health policies^(9)^. Therefore, it is essential to broaden discussions and research on the topic, creating a culture among professionals regarding continuity of care beyond the hospital.

The data obtained in the study are described and supported by Afaf Meleis’s Transitions Theory, considering the transition that dependent individuals and their caregivers undergo after an event that resulted in hospitalization and return home^(10)^.

## OBJECTIVES

To establish and implement the Hospital Discharge Management Committee (HDMC) in a university hospital, and to assesss the group’s perception of the committee’s planning and implementation.

## METHODS

### Ethical aspects

The parent project of which this study is a part respected the determinations of Resolutions 466/2012^(11)^ and 580/2018^(12)^ of the Brazilian National Health Council. It was submitted to and approved by the Research Ethics Committee, under Certificate of Presentation and Ethical Consideration.

The research ensured anonymity, preserving information confidentiality. Participants were identified by colors, informed of their right to opt out of the research and to withdraw from the study at any time, and then invited to sign the Informed Consent Form. Participants did not receive any financial benefit from their collaboration.

### Theoretical-methodological framework

The discussions in this study were based on Afaf Meleis’s Transitions Theory. According to this theory, transition is understood as a change from stable environments, conditions, or states, requiring individuals to have knowledge and a willingness to adapt internally and externally to the emerging paradigm^(13)^. This theory seeks to understand the nature of and responses to change in order to facilitate the experience, providing a framework that guides effective care before, during, and after transition. Thus, it guides professionals in preparing individuals undergoing transition processes to achieve mastery^(14)^.

### Study design

This is a qualitative study, developed through action research (AR), according to Michel Thiollent’s assumptions. He states that the relationships between the research device (AR), the observed situation (the context surrounding the problem), and the sphere of possible action (the location and its relationships) refer to actions that should be discussed, analyzed, and deliberated, valuing learning, mutual knowledge, and the outlining of decisions or strategies^(15)^. The study was structured according to the COnsolidated criteria for REporting Qualitative research (2007)^(16)^.

### Study setting

The study was conducted between February 2023 and February 2024 in a medium and high complexity university hospital in northeastern Brazil. In Brazil, up to the time of this study, only one university hospital had organized hospital discharge management^(17)^, although this is foreseen in Ordinance 3390^(18)^.

### Data source

Participants were professionals from the university hospital, selected through an internal call for applications for eight positions to form HDMC, organize, and establish it. Thus, the commission consisted of four nurses, three physiotherapists, and one social worker. Moreover, three nurses holding strategic positions in the hospital were invited: one from the Wound Care Commission, one from the Commission for Continuing Education in Nursing (CCEN), and one from the Bed Regulation and Management sector. Therefore, HDMC had 11 professionals. Two researchers who participated in the AR meetings and two undergraduate nursing students, recipients of scientific initiation scholarships, who organized the meetings, welcomed participants, distributed name tags, recorded interviews, and provided support as needed, also participated in the AR meetings.

### Data collection and organization

The hospital discharge management service organization began with the presentation of the proposal to the hospital’s CCEN, which suggested a permanent and multidisciplinary HDMC, initially serving a single unit as a pilot project.

The recommendation was shared with the hospital’s Nursing Division (ND), which contributed by suggesting the professional categories that should be included in the possible workload for the commission, as well as the number of participants. Following this, a meeting was arranged with the participation of the Healthcare Management, ND, CCEN, and research team members. At this meeting, the proposal was presented and discussed, the test unit was identified, and the creation and publication of a call for applications to recruit professionals to be part of HDMC was suggested.

The proposed pilot unit meets the following criteria: holding weekly multidisciplinary meetings and having a profile of inpatients with functional dependence. Therefore, the selected unit serves people with neurological and orthopedic diseases, with a prevalence of conditions such as stroke and multiple sclerosis, admitted through the State’s Bed Regulation Center or from the hospital’s own outpatient clinic. The unit has a multidisciplinary team and receives undergraduate nursing and physiotherapy students, as well as medical residents in neurology and orthopedics.

For the selection of professionals through a public notice, a panel was formed with two researchers and a nurse manager from the hospital, who considered, in the interview and curriculum analysis, the interest and affinity with the theme, manifested through a short text previously submitted, in addition to experiences related to home care, primary care, or continuity of care. It was understood that, to fulfill this role, it is necessary to have a perspective that goes beyond the hospital, considering the life context of people and the RAS.

### Work stages

The AR occurred in four phases: 1 - Diagnosis (to identify the problem); 2 - Action planning (actions to solve the problem); 3 - Action implementation (with selection of the action plan and implementation); 4 - Assessment (group perception of the planning of the meetings and HDMC implementation).

To strengthen the AR phases, a scoping review (INPLASY 202230143)^(19)^ was initially carried out to explore, in the literature, how hospital-to-home transitional care supports the home caregiver of older adults with functional dependence. This review supported the discussion in the AR meetings, when the data reported in the literature were presented and debated, especially in phase 1. Furthermore, the group discussions were enriched by participants’ care experience and by data related to a matrix project developed in the hospital in partnership with the university (School of Nursing), which accompanies people with dependence and caregivers in the hospital discharge process, supports the care team and performs telemonitoring up to 30 days after discharge.

### Data analysis

The analysis and discussion of AR results were based on Afaf Meleis’s Transitions Theory^(13)^.

## RESULTS

### Phase 1 - Situational diagnosis and action planning (diagnosis discussion, committee organization and establishment)

This phase was marked by three meetings (between April and May 2023). Initially, there were clarifications about the work objective, introduction of members, and agreements for the next meetings. Presentations and discussions took place regarding the of the scoping review, the master project, and the care practices developed at the hospital.

Subsequently, contributions to internal regulations were presented and discussed, leading to the establishment of HDMC through an ordinance published on May 22, 2023.

### Phase 2 - Action implementation planning and execution with the Hospital Discharge Management Committee

During the seven meetings of this phase (between July and October 2023), the internal regulations were approved, the president of the commission and the secretaries were identified, and a representative of the first discharge management service implemented in a university hospital in Brazil^(17)^ presented remotely, who spoke about the service, its structure and operation.

Also discussed and developed during this phase were the eligibility criteria, the recruitment and admission process for individuals eligible for follow-up by the committee, as well as the discharge and post-discharge follow-up flows. Although the need to manage the discharge of all individuals is recognized, HDMC suggested that, initially, criteria should be defined until a culture of follow-up for discharge is established among the hospital’s professionals.

The eligibility criteria were defined considering three aspects: level of dependence for nursing care according to the Fugulin scale^(20)^, validated in Brazil and applied to patients in the hospital wards daily by nurses; of the social support network situation; and formal support network situation. The instrument created by HDMC proposes a score ranging from one to 12, with a score of five being the cut-off point for follow-up (Figure 1).

**Figure 1:**
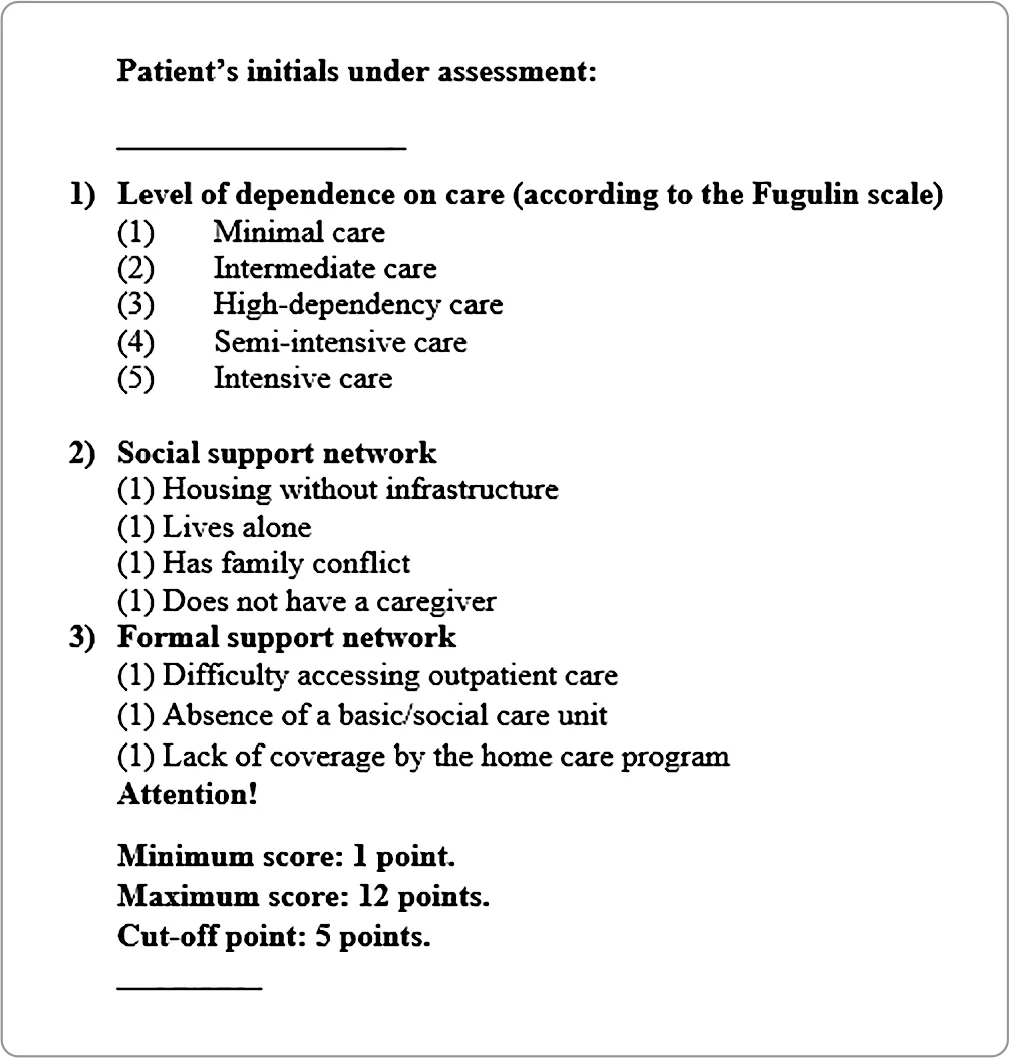
Eligibility criteria for patients for the Hospital Discharge Management Committee, Salvador, Bahia, Brazil, 2023

The patient recruitment and admission flow (Figure 2) aims to standardize the reception of individuals eligible for follow-up (following the eligibility criteria). According to the flow, these individuals will be recruited through weekly ward visits, by identifying new cases during weekly multidisciplinary discussions, or by referral from the care team via email, messaging app, or in person. When referred by the care team, HDMC must apply the eligibility criteria, verifying if the cut-off point is met.

**Figure 2:**
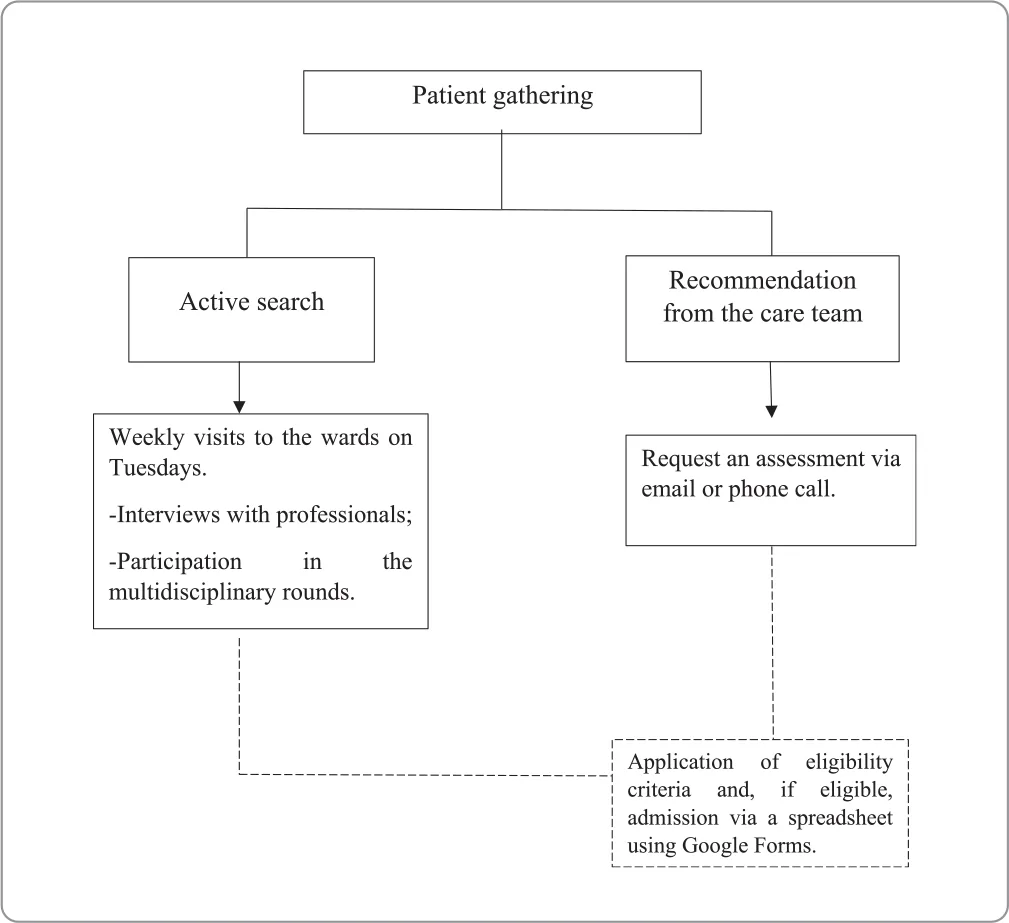
Patient recruitment flow for eligible patients by the Hospital Discharge Management Committee, Salvador, Bahia, Brazil, 2023

Meeting the eligibility criteria, HDMC conducts a biopsychosocial assessment to verify needs. Its role at this stage will involve: 1) communicating, transversally and periodically, needs for safe discharge to the unit team; 2) identifying potential interconsultations to expedite discharge; 3) developing a care plan, including needs assessment, monitoring, and supporting training provided to the care team.

Following the discharge follow-up flow (Figure 3), after admission, identification of problems and needs, the committee will develop a care plan to provide speed and safety at hospital discharge, supporting the care team in guidance, training, referrals, meetings with the family and whatever else is necessary.

**Figure 3:**
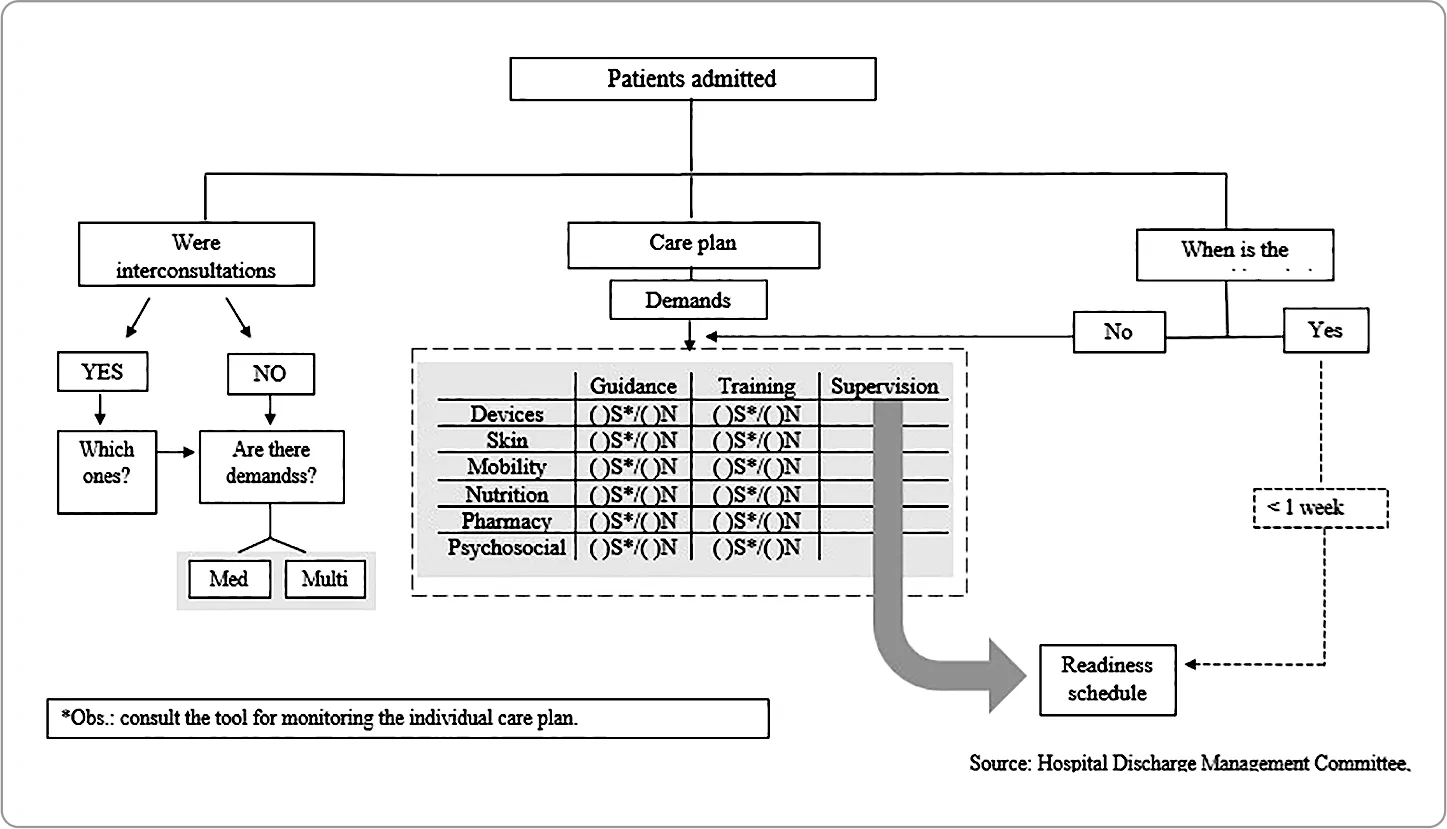
Patient follow-up flowchart for those admitted by the Hospital Discharge Management Committee, Salvador, Bahia, Brazil, 2023

Discharge interventions will also be supported by an instrument called the “*Instrumento de avaliação para a alta*” (Discharge assessment instrument), developed by HDMC to be applied upon admission and throughout the hospitalization. It indicates the needs of dependent person and/or their caregiver, assisting the team in actions for safe discharge through health guidance, social rights information, training, or supervision of any kind. Therefore, it becomes the central point of HDMC’s work process through self-reporting.

To this end, patients and/or caregivers are questioned and assessed in six aspects: handling of devices; skin care; mobility care; social support; provision of food; and medication use. For each aspect, self-report questions are applied, resulting in scores whose sum ranges from 18 to 54 points.

Thus, HDMC established a value equal to or below 43 points, which corresponds to 80% of the instrument’s score, to demonstrate the need for guidance/training and reinforcement in monitoring throughout hospitalization, postponement of discharge or, if postponement is not possible, home monitoring by telephone or video call for up to 30 days after returning to the community. If, after this period, the affected person and/or their family caregiver still require intervention regarding care, HDMC should discuss the possibility of referral to a service in the local care network. The discharge assessment instrument was created during the AR, based on the group’s care experience, scope review^(19)^ and data from the parent project from which this is taken. Currently, the instrument is in the process of content validity, and psychometric assessment will be carried out in the future.

The care plan developed for action throughout hospitalization involves guidance, training, and supervision as needed, addressing devices, skin, mobility, nutrition, medication use, and social support. In this regard, the committee describes necessary interventions based on the discharge assessment tool and follows up on patient and/or caregiver questions to ensure continuity of care, supporting the team.

This instrument will also be applied when discharge is already planned. If the intention is for discharge to occur in less than a week and there are still needs to be met for continuity of care, it is advisable to intensify guidance and training or postpone discharge so that patients acquire the necessary skills.

The workflows and instruments were tested through realistic simulation with a clinical case to apply the produced material, and, on that occasion, minor adjustments to the forms that should be applied were indicated.

### Phase 3 - Action implementation through the commission establishment

During this phase (between October and November 2023), three meetings were held to continue the implementation of the commission, putting into practice the flows and determinations of the documents. The material produced was disseminated at a meeting in the pilot unit and with other management authorities.

Also, during this phase, there was dialogue between HDMC and the hospital’s Humanization Committee, aiming at coordination and development of complementary actions. HDMC’s service schedule was developed with the inclusion of undergraduate research students.

### Phase 4 - Assessment of the committee’s perception of the Hospital Discharge Management Committee planning and implementation

At this stage, the group’s perception regarding the commission planning and implementation was assessed. This occurred procedurally, at the end of each meeting, and finally, after the last meeting, with HDMC implementation, following the initial follow-ups conducted with 16 patients and their caregivers, 40 days after the commission’s work began. In the procedural assessment, at the end of each meeting, participants were asked to freely assess the dynamics used and indicate, if necessary, changes for future meetings. These comments were recorded along with the recordings of all meetings.

For the final assessment, the aim was to understand the group’s perceptions after 40 days of implementing HDMC and its initial follow-ups. Therefore, a questionnaire was sent to the participants via a digital platform so they could express their perceptions of the changes in their work routine resulting from the AR. Only one participant did not respond to the survey, as they were on medical leave.

In a global context, all participants expressed satisfaction in participating as research members and approved of AR’s methodological choice for HDMC implementation and the construction of its work process. Furthermore, they reported feeling valued by participating in this committee, as evidenced by their statements:

*It broadened my perspective during my clinical practice to guide and prepare patients and their families for hospital discharge.* (Gray)

*It allowed me to refine my procedures and guidance in preparation for hospital discharge.* (Rose)

*In practice, I was able to identify our shortcomings and think about how our processes could contribute to the service, patients, and their families.* (Purple)

In this immediate phase, the aim was to assess the group’s perception regarding HDMC planning and implementation. Hence, the group perceived it as satisfactory, improving their practice with patients and broadening their perspective on the relevance of this topic. This positive perception from the group is important so that they can continue the commission beyond the research phase. Currently, an assessment of its impact on the people being monitored is underway.

## DISCUSSION

In phase 1, the group identified the care model best suited to the profile of the patients served, as well as the tools necessary to improve discharge management at the university hospital. Discharge management presents important elements to promote continuity of care, configuring itself in the triad of management, education, and care, in pursuit of comprehensive, safe, quality healthcare directed at meeting the user’s needs^(21)^.

Chronic illness requires patients and caregivers to learn various skills to cope with changing roles and adapt to new health conditions and care demands. According to Meleis^(10)^, transition processes can cause changes in identity and roles, making individuals vulnerable to the impact on their life. In this scenario, discharge management should be viewed as a longitudinal phenomenon that, starting at admission, involves discharge planning and extends beyond discharge, in order to improve quality of care outcomes^(22)^.

In the literature, nurses appear as one of the most involved professionals, coordinating discharge management for continuity of care^(8)^. Beyond this professional, authors point to the importance of multidisciplinary planning for hospital discharge, fully addressing patients’ needs and ensuring more assertive and safe care^(2,23)^. Seeking to build collaborative work, with the articulation and integration of knowledge, HDMC was conceived as a multidisciplinary team, composed of nurses, physiotherapists and a social worker, coordinated by a nurse.

With the development of eligibility criteria, patient recruitment and admission flows, and discharge and post-discharge follow-up flows (Phase 2), the committee implemented its work process. The creation of care support tools introduces technologies that simplify and optimize care processes for continuity of care, facilitating the identification of patients for follow-up by HDMC and promoting a smoother transition between the hospital and the community.

The capacity or ability to develop new skills related to basic care is essential for successfully completing the transition. Therefore, assessment for safer continuity of care can be carried out based on individual response patterns^(10)^, called process and outcome indicators, which allow identifying whether the person is moving towards health and well-being or towards vulnerability and risks. To this end, HDMC developed the discharge assessment instrument with the aim of monitoring, through self-report, the acquisition of skills and the need for guidance and training, aiming to continue care and enable the achievement of mastery. This assessment is important to guide discharge planning, as this is a moment of vulnerability for the person and their caregiver, characterized by changes involving new needs and responsibilities for care and management^(24)^.

In this same context, a Brazilian study constructed and validated a checklist for preparing patients for discharge, using 17 items considered essential for a safe discharge that involved social support, care, and guidance regarding procedures related to home care and referrals to other services^(2)^. At HDMC, the creation of the discharge assessment instrument aimed to guide the team’s conduct in planning interventions, facilitating communication between members, the care team, and patients/caregivers, standardizing an assessment to guide the care team regarding continuity of care.

HDMC implementation at the university hospital aims to improve the follow-up of hospitalized individuals, most of whom are socially vulnerable and live in rural areas. This will facilitate communication with the RAS to reduce length of stay and expand intervention strategies through collective planning and post-discharge monitoring with shared care. Beyond addressing health needs, HDMC recognizes that the follow-up flow facilitates the control of administrative issues involved in hospitalization, such as waiting for multidisciplinary consultations, undergoing examinations, completing standard forms necessary for care follow-up, activating other network resources, etc., which can delay follow-up or even prevent patient discharge.

Regarding post-discharge monitoring, the committee understood systematic and multidisciplinary follow-up as a complementary strategy to assist in home adaptation. Structured post-discharge monitoring protocols have been used to review self-care guidelines, maintenance, health promotion, and referral to the health network^(4)^. Based on this, HDMC developed a tool to monitor people for up to 30 days after discharge, the period of greatest risk for avoidable readmissions. In southeastern Brazil, 40.4% of patients with chronic diseases had unplanned readmissions within 30 days of discharge^(25)^. This reinforces the need to monitor the adaptation process to minimize adverse events and other complications.

Discharge management models in Spain and Portugal include caregivers during the planning for continuity of care^(8,7)^. However, given the profile of the people treated at the university hospital, meeting with the responsible caregivers is not always possible, as many are from other municipalities and even rural areas, alternating care with family members, friends, or neighbors. Thus, it is necessary to consider strategies for the inclusion of these people, considering the importance of supporting them in carrying out this function. Caregivers’ involvement in care planning offers security, allowing their participation in decisions for the development and execution of a therapeutic plan articulated to the life context^(26)^, in the face of the intense transitions caused by the health-disease process^(10)^.

Academics’ contribution to this committee was significant, especially in health education and post-discharge follow-up. Experience in a university hospital in Paraná with nursing residents under the preceptorship of liaison nurses highlights the importance of academic involvement in the development of management activities, technical visits, and case discussions with the team^(1)^. Integration into HDMC enhances students’ skills and abilities, promoting integration between teaching and service and building active and reflective learning^(27)^. Therefore, this discharge management model can be used in other teaching hospitals after adjustments to different realities.

When assessing the group’s perception of HDMC planning and implementation, it was identified that the AR favored the collective construction of the commission, mediating points that could hinder the process and jointly finding solutions to difficulties. In this sense, the AR offers means for members to reflect on and plan intentional actions that lead to better conditions and processes in their own practice^(28)^. Thus, the AR fulfilled its function of changing a given reality, providing benefits to those involved and to the service^(29)^. Furthermore, it touched the group regarding the relevance of this topic, motivating them to move forward.

### Study limitations

One limitation is the short time available to monitor the impact of the commission’s actions on improving discharge planning at the university hospital.

### Contributions to nursing and health

This research made it possible to establish and implement a committed and coordinated HCMS within the service. The results of this research contribute to the qualification of nursing services and other healthcare professionals, aiming at preparing patients and families for continuity of care at home. Furthermore, it contributes to safe and rapid discharge and to the reduction of preventable adverse events at home that are responsible for readmissions.

## FINAL CONSIDERATIONS

The study established and implemented HDMC at the university hospital and assessed the group’s perception of HDMC planning and implementation for monitoring highly dependent individuals and their caregivers. To this end, it developed instruments and workflows such as eligibility criteria, patient recruitment and admission flow, a readiness scale, and follow-up, discharge, and post-discharge flows, building a model to support patients and caregivers in continuity of care.

The group’s perception was positive, both regarding the method used and the theme, as well as the impact of the commission on their practices. This is important for continuity of HDMC. The involvement of professionals from different categories broadened the perspective on the needs for intervention and guidance in health for patients and their families. The feeling of belonging to the collective was strengthened through the appreciation of each individual contribution and through theoretical empowerment regarding the theme to be discussed and worked on within the institution.

The workflows and tools developed will need to be validated in the future so that they can be replicated in other care settings, and an assessment of the impact of HDMC on the patients monitored will also be conducted.

This result serves as a model for other initiatives, especially in hospitals that receive students. HDMC at the university hospital aims for its development and expansion to all units of the hospital, in addition to coordination with the Internal Regulation Center, implemented after the completion of this AR.

## Data Availability

The research data are available within the article.
