# Supplementary material for: Implementation of patient discharge management in a university hospital through action research
Source: Rev Bras Enferm. 2026 Oct 19;79:e20250174. doi: 10.1590/0034-7167-2025-0174 (PMC13679592; doi:10.1590/0034-7167-2025-0174)
Supplement: Translation

# Translation: Implantação da gestão da alta do paciente em hospital universitário através de pesquisa-ação

**DOI:** 10.1590/0034-7167-2025-0174pt

**Authors:** Lélia Mendes Sobrinho de Oliveira, Larissa Chaves Pedreira, Ana Paula Santos de Jesus, Elizabeth Bernardino, Cristina Rosa Soares Lavareda Baixinho, Valdenir Almeida da Silva, Amanda Aparecida Silva Rios, Emanuela Santos Oliveira

**Affiliations:** I Universidade Federal da Bahia. Salvador, Bahia, Brasil; II Universidade do Recôncavo Baiano. Cruz das Almas, Bahia, Brasil; III Universidade Federal do Paraná. Curitiba, Paraná, Brasil; IV Escola Superior de Enfermagem de Lisboa. Lisboa, Portugal

**Keywords:** Cuidado Transicional, Gestão da Segurança, Pesquisa-Ação, Hospital Universitário, Alta do Paciente.

## Abstract

**Objetivos:**

instituir e implantar a Comissão de Gestão da Alta Hospitalar em hospital universitário, e avaliar a percepção do grupo sobre o planejamento e implantação da comissão.

**Métodos:**

estudo qualitativo, realizado através de pesquisa-ação. Ocorreu em hospital universitário do Nordeste brasileiro, durante 12 meses, seguindo quatro fases: diagnóstico situacional; planejamento das ações; implementação das ações; e avaliação.

**Resultados:**

a comissão foi instituída e implantada no hospital, com processo de trabalho construído coletivamente. Elaboraram-se instrumentos e fluxos como critérios de elegibilidade, captação e admissão de pacientes, escala de prontidão, além dos fluxos de acompanhamento, alta e pós-alta, estruturando um modelo de apoio à continuidade do cuidado.

**Considerações Finais:**

a comissão foi implantada para acompanhar pacientes na unidade piloto. Na percepção do grupo, houve melhora no acompanhamento de pacientes e cuidadores, além de avaliação positiva da formação da comissão e da metodologia utilizada, valorizando cada contribuição individual.

## INTRODUÇÃO

O gerenciamento da alta hospitalar é uma ferramenta administrativa utilizada na promoção de intervenções efetivas para integrar pontos da Rede de Atenção à Saúde (RAS), garantindo a continuidade do cuidado^(1)^. Envolve planejamento e estruturação da alta, além de orientação de familiares e cuidadores, identificação e articulação com a RAS^(2)^. Essa discussão é necessária, principalmente, em países desenvolvidos e em desenvolvimento, devido ao envelhecimento da população e maior dependência funcional dessa faixa etária, especialmente após longos períodos de internamento hospitalar.

No Brasil, a Política Nacional de Atenção Hospitalar^(3)^ pontua sobre a alta hospitalar responsável através da transferência dos cuidados. A despeito disso, esse debate é recente no país. Na prática clínica, observam-se poucas ações sobre o tema, especialmente na rede pública, onde a população mais precisa. Ademais, essa lacuna também é relatada por autores nacionais^(4,5)^, necessitando de intensificação, especialmente no contexto da população vulnerável, de baixa renda e nível educacional, com pouco suporte formal ou social.

A falta de planejamento adequado para alta hospitalar de pessoas em situação de vulnerabilidade, que envolve a continuidade dos cuidados, está relacionada a efeitos adversos graves como erros de medicação, atrasos no cuidado, duplicidade do tratamento, redução da adesão, piora na qualidade de vida da pessoa e seu cuidador, readmissões evitáveis, uso inadequado dos serviços, e aumento do custo em saúde^(4)^. Logo, a gestão da alta hospitalar facilita a continuidade dos cuidados e o uso eficiente dos recursos hospitalares e comunitários, a partir da identificação e atuação nas necessidades do paciente e seu cuidador, considerando contextos de vida^(5)^.

Experiências internacionais com a gestão da alta para a continuidade do cuidado têm sido relatadas em países como Espanha^(6)^, Portugal^(7)^ e Canadá^(8)^. No Brasil, estudo realizado com profissionais da equipe multidisciplinar para elaboração e validação de *checklist* objetivando organizar a alta hospitalar indica resultados positivos após um trabalho coordenado para elaborar planos de alta multiprofissionais conforme as necessidades dos pacientes e responsáveis. Os autores enfatizam que o resultado pode ser mais satisfatório quando os planos de alta são articulados entre os diferentes níveis de atenção à saúde^(2)^.

Nesse contexto, os enfermeiros se destacam na coordenação e desenvolvimento da continuidade do cuidado, tendo em vista suas habilidades e competências gerenciais, de educação em saúde, além da articulação com demais profissionais da equipe e maior tempo em contato com pacientes e familiares. Além disso, possuem conhecimento dos sistemas e serviços de saúde disponíveis para o seguimento da assistência, tornando importante o aprofundamento do estudo do cuidado de transição e o potencial de atuação enquanto membro da equipe multiprofissional^(1,4,8)^.

Com isso, urge a necessidade de implementar estratégias para garantir uma transição segura entre o hospital e o domicílio, aprimorando planos de alta multiprofissionais nos processos de trabalho, bem como nas políticas de saúde do país^(9)^. Para tanto, é fundamental ampliar discussões e pesquisas sobre o tema, criando uma cultura entre os profissionais sobre a continuidade do cuidado para além do hospital.

Os dados logrados no estudo estão descritos e foram embasados pela Teoria das Transições de Afaf Meleis, considerando a transição pela qual pessoas dependentes de cuidados e seus cuidadores passam após um evento que gerou hospitalização e retorno domiciliar^(10)^.

## OBJETIVOS

Instituir e implantar a Comissão de Gestão da Alta Hospitalar (CGAH) em hospital universitário, e avaliar a percepção do grupo sobre o planejamento e implantação da comissão.

## MÉTODOS

### Aspectos éticos

O projeto matriz do qual este estudo faz parte respeitou as determinações das Resoluções nº 466/2012^(11)^ e nº 580/2018^(12)^ do Conselho Nacional de Saúde. Foi submetido e aprovado pelo Comitê de Ética em Pesquisa, sob Certificado de Apresentação e Apreciação Ética.

A pesquisa garantiu o anonimato, preservando o sigilo das informações. Os participantes foram nomeados por cores, informados sobre seu direito de optar por participar ou não da pesquisa e de desistir do estudo a qualquer momento, e então convidados a assinar o Termo de Consentimento Livre e Esclarecido. Os participantes não tiveram nenhum benefício financeiro com a sua colaboração.

### Referencial teórico-metodológico

As discussões deste estudo tiveram como base a Teoria da Transição proposta por Afaf Meleis. Para ela, a transição é entendida como a mudança de ambientes, condições ou estados estáveis, exigindo do indivíduo conhecimento e disposição para adaptações internas e externas diante do paradigma emergente^(13)^. Essa teoria busca compreender a natureza e as respostas à mudança para facilitar a experiência, fornecendo uma estrutura que orienta o cuidado eficaz antes, durante e após a transição. Assim, orienta os profissionais no preparo de pessoas em processos de transição para aquisição da maestria^(14)^.

### Tipo de estudo

Trata-se de estudo qualitativo, desenvolvido por meio da pesquisa-ação (PA), conforme pressupostos de Michel Thiollent. Ele afirma que as relações entre o dispositivo de investigação (PA), a situação observada (o contexto que envolve o problema) e a esfera da ação possível (o local e as suas relações) remetem às ações que devem ser discutidas, analisadas e deliberadas, valorizando aprendizagem, conhecimento mútuo e delineamento de decisões ou estratégias^(15)^. O estudo foi estruturado conforme referência do *COnsolidated criteria for REporting Qualitative research* (2007)^(16)^.

### Cenário do estudo

Realizou-se entre fevereiro de 2023 e fevereiro de 2024 em hospital universitário de média e alta complexidade do Nordeste brasileiro. No Brasil, até o momento deste estudo, apenas um hospital universitário possuía gestão da alta hospitalar organizada^(17)^, embora esta seja prevista na Portaria nº 3390^(18)^.

### Fonte de dados

Os participantes foram profissionais do hospital universitário, selecionados através de edital interno com oito vagas para compor a CGAH, realizar a sua organização e instituí-la. Assim, constituíram a comissão quatro enfermeiros, três fisioterapeutas e um assistente social. Além disso, foram convidados três enfermeiros que ocupam cargos estratégicos no hospital: um da Comissão de Feridas, um da Comissão de Educação Permanente em Enfermagem (CEPE) e um do setor de Regulação e Gestão de Leitos. Com isso, a CGAH contou com 11 profissionais. Participaram ainda dos encontros da PA duas pesquisadoras que conduziram as oficinas e duas graduandas de enfermagem, bolsistas de iniciação científica, que organizaram os encontros, receberam os participantes, distribuíram crachás, gravaram as entrevistas e apoiaram no que fosse necessário.

### Coleta e organização dos dados

A organização do serviço de gestão da alta hospitalar teve início com a apresentação da proposta para o CEPE do hospital, que sugeriu uma CGAH permanente e multiprofissional, atendendo inicialmente uma única unidade como piloto.

A recomendação foi socializada com a Divisão de Enfermagem (DE) do hospital, que contribuiu sugerindo as categorias profissionais que deveriam integrar a carga horária possível para trabalho na comissão, bem como o número de participantes. A partir daí, foi oportunizada uma reunião com a participação da Gerência de Atenção à Saúde, DE, CEPE e membros da pesquisa. Nesse encontro, foi apresentada e discutida a proposta, identificando-se a unidade teste e sugerindo-se a confecção e publicação de edital para captação dos profissionais que fariam parte da CGAH.

A unidade piloto sugerida atende aos seguintes critérios: realizar reuniões multiprofissionais semanais e ter um perfil de pacientes internados com dependência funcional. Logo, a unidade selecionada atende pessoas com doenças neurológicas e ortopédicas, com prevalência de situações como acidente vascular cerebral e esclerose múltipla, admitidos via Central de Regulação de Leitos do Estado ou oriundas do ambulatório do próprio hospital. A unidade possui equipe multiprofissional e recebe graduandos de enfermagem e fisioterapia, além de residentes médicos de neurologia e ortopedia.

Para seleção dos profissionais através de edital, formou-se uma banca com duas pesquisadoras e um enfermeiro gestor do hospital, que consideraram, na entrevista e na análise do currículo, o interesse e a afinidade com o tema, manifestados por meio de um pequeno texto enviado previamente, além de experiências relacionadas à atenção domiciliar, à atenção primária ou à continuidade do cuidado. Compreendeu-se que, para exercer esse papel, é necessário ter um olhar que vá além do hospital, considerando o contexto de vida das pessoas e a RAS.

### Etapas do trabalho

A PA ocorreu em quatro fases: 1 - Diagnóstico (para identificar o problema); 2 - Planejamento das ações (ações para resolver o problema); 3 - Execução das ações (com seleção do roteiro de ação e implementação destas); 4 - Avaliação (percepção do grupo sobre o planejamento dos encontros e a implantação da CGAH).

Para fortalecer as fases da PA, realizou-se, inicialmente, revisão de escopo (INPLASY 202230143)^(19)^ objetivando explorar, na literatura, como o cuidado transicional hospital-domicílio apoia o cuidador domiciliar de pessoas idosas com dependência funcional. Esta revisão apoiou a discussão nos encontros da PA, quando os dados reportados na literatura foram apresentados e debatidos especialmente na fase 1. Além disso, as discussões do grupo foram enriquecidas pela experiência assistencial das participantes e pelos dados relacionados a um projeto matriz desenvolvido no hospital em parceria com a universidade (Escola de Enfermagem), que acompanha pessoas com dependência e cuidadores no processo de alta hospitalar, apoia a equipe assistencial e realiza telemonitoramento até 30 dias após a alta.

### Análise dos dados

A análise e a discussão dos resultados da PA tiveram como base a Teoria das Transições proposta por Afaf Meleis^(13)^.

## RESULTADOS

### Fase 1 - Diagnóstico situacional e planejamento da ação (discussão do diagnóstico, organização e instituição da comissão)

Esta fase foi marcada por três encontros (entre abril e maio de 2023). Inicialmente, houve esclarecimentos sobre o objeto de trabalho, apresentação dos membros e pactuações para os próximos encontros. Ocorreram apresentação e discussão dos resultados da revisão de escopo, do projeto matriz e das práticas assistenciais desenvolvidas no hospital.

Posteriormente, foram apresentadas e discutidas as contribuições ao regimento interno, instituindo-se a CGAH através de portaria publicada em 22 de maio de 2023.

### Fase 2 - Planejamento e execução da ação com a implantação da Comissão de Gestão da Alta Hospitalar

No transcorrer dos sete encontros desta fase (entre julho e outubro de 2023), ocorreram a aprovação do regimento interno, identificação do presidente da comissão e dos secretários, além da apresentação, de forma remota, de representante do primeiro serviço de gestão da alta implantado em hospital universitário do Brasil^(17)^, que falou sobre o serviço, sua estrutura e funcionamento.

Foram também discutidos e construídos nesta fase os critérios de elegibilidade, o fluxo de captação e admissão de pessoas elegíveis ao acompanhamento pela comissão, bem como os fluxos de acompanhamento de alta e pós-alta. Embora se reconheça a necessidade de realizar a gestão da alta de todas as pessoas, a CGAH sugeriu que, a princípio, deveriam ser definidos critérios até que a cultura do acompanhamento para a alta se instalasse entre os profissionais do hospital.

Os critérios de elegibilidade foram definidos considerando três aspectos: nível de dependência para os cuidados de enfermagem conforme escala de Fugulin^(20)^, validada no Brasil e aplicada aos pacientes das enfermarias do hospital diariamente pelos enfermeiros; situação da rede de apoio social; e situação da rede de apoio formal. O instrumento criado pela CGAH propõe um escore variando entre um e 12, sendo a pontuação cinco o ponto de corte para acompanhamento (Figura 1).

**Figura 1:** Critérios de elegibilidade dos pacientes para a Comissão de Gestão da Alta Hospitalar, Salvador, Bahia, Brasil, 2023

O fluxo de captação e admissão de pacientes (Figura 2) objetiva padronizar a recepção das pessoas elegíveis ao acompanhamento (seguindo os critérios de elegibilidade). Conforme o fluxo, estas serão captadas através de visitas semanais nas enfermarias, por identificação de novos casos durante as discussões multiprofissionais semanais ou por indicação da equipe assistencial através de e-mail, aplicativo de mensagem ou presencialmente. Quando indicadas pela equipe assistencial, a CGAH deve aplicar os critérios de elegibilidade, verificando se atinge o ponto de corte.

**Figura 2:** Fluxo de captação de pacientes elegíveis pela Comissão de Gestão da Alta Hospitalar, Salvador, Bahia, Brasil, 2023

Atendendo aos critérios de elegibilidade, a CGAH realiza anamnese biopsicossocial para verificar demandas. A atuação desta, neste momento, envolverá: 1) sinalização para a equipe da unidade, de forma transversal e periódica, sobre demandas para a alta segura; 2) identificação de possíveis interconsultas para agilizar a alta; 3) elaboração do plano de cuidados, com o levantamento das necessidades, acompanhamento e apoio aos treinamentos realizados pela equipe assistencial.

Seguindo o fluxo de acompanhamento para a alta (Figura 3), após admissão, identificação dos problemas e demandas, a comissão elaborará um plano de cuidados para oferecer agilidade e segurança na alta hospitalar, apoiando a equipe assistencial nas orientações, treinamentos, encaminhamentos, reuniões com a família e no que mais for necessário.

**Figura 3:** Fluxo de acompanhamento dos pacientes admitidos pela Comissão de Gestão da Alta Hospitalar, Salvador, Bahia, Brasil, 2023

As intervenções para a alta serão apoiadas também por um instrumento denominado “Instrumento de avaliação para a alta”, construído pela CGAH para ser aplicado na admissão e ao longo do internamento. Ele indica as demandas da pessoa com dependência e/ou do seu cuidador para o cuidado, auxiliando a equipe nas ações para a alta segura, por meio de orientações em saúde, direitos sociais, treinamentos ou supervisão de qualquer natureza. Logo, torna-se o ponto central do processo de trabalho da CGAH através do autorrelato.

Para isso, pacientes e/ou cuidadores são questionados e avaliados em seis aspectos: manuseio de dispositivos; realização de cuidados com a pele; realização de cuidados com a mobilidade; apoio social; oferta da alimentação; e uso de medicamentos. Para cada aspecto, são aplicadas questões de autorresposta que configuram pontuações cuja soma varia entre 18 e 54 pontos.

Assim, foi estabelecido pela CGAH o valor igual ou abaixo de 43 pontos, que corresponde a 80% na pontuação do instrumento, para comprovar a necessidade de orientações/treinamentos e de reforço no acompanhamento ao longo da internação, postergação da alta ou, caso não seja possível postergar, de acompanhamento no domicílio por telefone ou videochamada em até 30 dias após o retorno à comunidade. Se, após esse período, a pessoa acometida e/ou seu cuidador familiar ainda apresentar necessidade de intervenção com relação ao cuidado, deverá ser discutida pela CGAH a possibilidade de encaminhamento para algum serviço da rede de assistência local. O instrumento de avaliação para a alta foi criado durante a PA, a partir da experiência assistencial do grupo, da revisão de escopo^(19)^ e dos dados do projeto matriz do qual este é recorte. No momento, o instrumento está em processo de validação do seu conteúdo, e, futuramente, será realizada a avaliação psicométrica.

O plano de cuidados construído para ação ao longo do internamento envolve orientação, treinamento e supervisão conforme demanda, direcionados aos dispositivos, à pele, à mobilidade, à alimentação, ao uso de medicamentos e ao apoio social. Nesse sentido, a comissão descreve intervenções necessárias com base no instrumento de avaliação para a alta e acompanha as dúvidas dos pacientes e/ou cuidadores para assegurar a continuidade dos cuidados, apoiando a equipe.

Este instrumento também será aplicado quando a alta já está prevista. Se a pretensão é que esta ocorra em menos de uma semana e ainda há demandas a serem atendidas para a continuidade do cuidado, deve-se indicar a intensificação de orientações e treinamentos ou a postergação da alta, para que o paciente adquira as habilidades necessárias.

Os fluxos e instrumentos foram testados através de simulação realística com caso clínico para aplicação do material produzido, e, na ocasião, sinalizou pequenas adequações nos formulários que deveriam ser aplicados.

### Fase 3 - Execução da ação através da implantação da comissão

Nesta fase (entre outubro e novembro de 2023), houve três encontros para dar continuidade à implantação da comissão, colocando em prática os fluxos e determinações dos documentos. O material construído foi divulgado em reunião na unidade piloto e com demais autoridades da gestão.

Ainda nessa fase, houve a interlocução entre a CGAH e a Comissão de Humanização do hospital, visando articulação e desenvolvimento de ações complementares. Desenvolveu-se a escala de serviço da CGAH com a inclusão dos estudantes de iniciação científica.

### Fase 4 - Avaliação de percepção da comissão sobre o planejamento e implantação da Comissão de Gestão da Alta Hospitalar

Nesta fase, avaliou-se a percepção do grupo com relação ao planejamento e implantação da comissão. Ocorreu de forma processual, no final de cada encontro, e final, após o último encontro, com a implantação da CGAH, após os primeiros acompanhamentos, realizados com 16 pacientes e seus cuidadores, transcorridos 40 dias após o início dos trabalhos da comissão. Na avaliação processual, ao final de cada encontro, solicitou-se aos integrantes que avaliassem livremente a dinâmica utilizada e indicassem, caso necessário, alterações para os próximos momentos. Estas falas foram gravadas junto com a gravação de todos os encontros.

Para a avaliação final, buscou-se compreender as percepções do grupo após 40 dias de implantação da CGAH e seus primeiros acompanhamentos. Assim, foi encaminhado questionário aos participantes, via plataforma digital, para que pudessem expressar as percepções das mudanças na sua rotina de trabalho a partir da PA. Apenas um integrante não respondeu ao inquérito, por se encontrar em licença médica.

Em um contexto global, todos os participantes manifestaram satisfação em participar como membro da pesquisa e aprovaram a escolha metodológica da PA para a implantação da CGAH e construção do seu processo de trabalho. Ademais, relataram se sentir valorizados participando desta comissão, conforme relatos:

*Ampliou o olhar durante a minha prática clínica para orientar e preparar os pacientes e familiares para a alta hospitalar*. (Cinza)

*Possibilitou que pudesse aperfeiçoar minhas condutas e orientações visando à alta hospitalar*. (Rosa)

*Na prática, pude perceber nossos pontos falhos e pensar em como nossos fluxos poderão contribuir para o serviço, pacientes e seus familiares*. (Roxo)

Nessa fase imediata, buscou-se avaliar a percepção do grupo com relação ao planejamento e implantação da CGAH. Nesse sentido, o grupo percebeu esta como satisfatória, melhorando a sua prática junto aos pacientes e ampliando o olhar com relação à relevância desse tema. Essa percepção positiva do grupo é importante para que possam dar continuidade à comissão para além da pesquisa. No momento, está em curso uma avaliação do impacto desta nas pessoas acompanhadas.

## DISCUSSÃO

Na fase 1, o grupo identificou o modelo assistencial mais adequado ao perfil dos pacientes assistidos, bem como as ferramentas necessárias para a qualificação da gestão da alta no hospital universitário. A gestão da alta apresenta elementos importantes para promover a continuidade do cuidado, configurando-se na tríade gestão, educação e assistência, em busca de uma atenção em saúde integral, segura, de qualidade e direcionada a atender às necessidades do usuário^(21)^.

O adoecimento crônico requer do paciente e cuidadores aprendizados diversos para lidar com as mudanças de papéis e adaptar-se às novas condições de saúde e exigências de cuidado. Conforme Meleis^(10)^, os processos de transição podem causar mudanças de identidade e papéis, tornando o indivíduo vulnerável a depender do impacto em sua vida. Nesse cenário, a gestão da alta deve ser encarada como um fenômeno longitudinal que, iniciado na admissão, envolve o planejamento da alta e se estende após esta, a fim de melhorar a qualidade dos resultados assistenciais^(22)^.

Na literatura, o enfermeiro aparece como um dos profissionais mais envolvidos, coordenando a gestão da alta para a continuidade do cuidado^(8)^. Para além desse profissional, autores apontam a importância do planejamento multiprofissional para a alta hospitalar, atendendo integralmente às necessidades do paciente e garantindo uma assistência mais assertiva e segura^(2,23)^. Buscando a construção de um trabalho colaborativo, com articulação e integração de conhecimentos, a CGAH foi concebida como uma equipe multidisciplinar, composta por enfermeiros, fisioterapeutas e assistente social, coordenada pelo enfermeiro.

Com a elaboração dos critérios de elegibilidade, fluxo de captação, e admissão dos pacientes e fluxos de acompanhamento de alta e após a alta (Fase 2), a comissão implantou o seu processo de trabalho. A criação dos instrumentos de apoio ao cuidado introduz tecnologias que simplificam e otimizam os processos assistenciais para a continuidade do cuidado, facilitando a identificação de pacientes para acompanhamento pela CGAH e promovendo uma transição mais fluida entre o hospital e a comunidade.

A capacidade ou habilidade para desenvolver novas competências relacionadas aos cuidados básicos é imprescindível para cumprir a transição com sucesso. Dessa maneira, a avaliação para a continuidade mais segura do cuidado pode ser realizada a partir de padrões de resposta do indivíduo^(10)^, denominados indicadores de processos e de resultados, que permitem identificar se a pessoa se encontra na direção de saúde e bem-estar ou na direção de vulnerabilidade e riscos. Para isso, a CGAH elaborou o instrumento de avaliação para a alta com o objetivo de monitorar, através do autorrelato, a aquisição de habilidades e a necessidade de orientação e treinamento, visando continuar o cuidado e possibilitar o alcance da maestria. Essa avaliação é importante para orientar o planejamento da alta, pois esse é um momento de vulnerabilidade para a pessoa e seu cuidador, caracterizado por mudanças que envolvem novas necessidades e responsabilidades de cuidado e gestão^(24)^.

Nesse mesmo contexto, estudo brasileiro construiu e validou *checklist* para o preparo da alta de pacientes, utilizando 17 quesitos considerados indispensáveis para alta segura que envolviam suporte social, cuidados e orientações quanto aos procedimentos ligados à assistência em domicílio e aos encaminhamentos para outros serviços^(2)^. Na CGAH, a criação do instrumento de avaliação para alta buscou nortear a conduta da equipe no planejamento de intervenções, facilitando a comunicação entre os membros, equipe assistencial e pacientes/cuidadores, padronizando uma avaliação para orientar a equipe assistencial com relação à continuidade do cuidado.

A implantação da CGAH no hospital universitário intenciona melhorar o acompanhamento das pessoas internadas, maioria em vulnerabilidade social e vivendo na zona rural, favorecendo a interlocução com a RAS para reduzir o tempo de internação e ampliar as estratégias de intervenção através de planejamento coletivo e monitoramento após a alta com cuidados compartilhados. Além da atenção às necessidades em saúde, a CGAH compreende que o fluxo de acompanhamento favorece o controle das questões administrativas envolvidas no internamento, como a espera por interconsultas multidisciplinares, a realização de exames, o preenchimento de impressos padrão necessários ao seguimento assistencial, o acionamento de outros dispositivos da rede, *etc*., que podem atrasar o acompanhamento ou até mesmo inviabilizar a alta do paciente.

Sobre o monitoramento após a alta, a comissão compreendeu o acompanhamento sistematizado e multidisciplinar como uma estratégia complementar para auxiliar na adaptação em domicílio. Roteiros estruturados de monitoramento após a alta vêm sendo utilizados para revisar orientações de autocuidado, manutenção, promoção da saúde e direcionamento à rede de saúde^(4)^. Baseado nisso, a CGAH construiu um instrumento para acompanhar as pessoas até 30 dias após a alta, período de maior risco para readmissões evitáveis. No Sudeste brasileiro, 40,4% dos pacientes com doenças crônicas tiveram readmissões não planejadas em até 30 dias após a alta^(25)^. Isso reforça a necessidade do acompanhamento do processo de adaptação para minimizar eventos adversos e demais complicações.

Modelo de gestão da alta na Espanha e em Portugal inclui os cuidadores durante o planejamento para continuidade do cuidado^(8,7)^. Entretanto, pelo perfil das pessoas atendidas no hospital universitário, o encontro com os cuidadores responsáveis nem sempre é possível, pois grande parte são de outro município e até de zona rural, revezando o acompanhamento com pessoas da família, amigos ou vizinhos. Assim, é preciso pensar estratégias para a inclusão dessas pessoas, considerando a importância de apoiá-los para o exercício dessa função. O envolvimento dos cuidadores no planejamento dos cuidados oferece segurança, permitindo a sua participação nas decisões para desenvolvimento e execução do plano terapêutico articulado ao contexto de vida^(26)^, diante das intensas transições ocasionadas pelo processo de saúde-doença^(10)^.

A contribuição de acadêmicos nesta comissão foi relevante, especialmente na educação em saúde e nos acompanhamentos após a alta. Experiência em hospital universitário no Paraná com residentes de enfermagem sob a preceptoria da enfermeira de ligação relata a importância do envolvimento dos acadêmicos no desenvolvimento das atividades de gerenciamento, visitas técnicas e discussão de casos com a equipe^(1)^. A inserção na CGAH aprimora as competências e habilidades dos estudantes, favorecendo a integração entre ensino e serviço e construindo uma aprendizagem ativa e reflexiva^(27)^. Assim, este modelo de gestão da alta pode ser utilizado em outros hospitais de ensino após ajustes a realidades distintas.

Na avaliação da percepção do grupo sobre o planejamento e implantação da CGAH, identificou-se que a PA favoreceu a construção da comissão de forma coletiva, mediando pontos que poderiam dificultar o processo e encontrando conjuntamente solução para as dificuldades. Nesse sentido, a PA oferece meios para os membros refletirem e planejarem ações intencionais que conduzam a melhores condições e processos da sua própria prática^(28)^. Assim, a PA cumpriu a sua função de mudar determinada realidade, proporcionando benefício aos envolvidos e ao serviço^(29)^. Ademais, tocou o grupo com relação à relevância deste tema, motivando-o a seguir adiante.

### Limitações do estudo

Como limitação, aponta-se o curto tempo disponível para acompanhamento do impacto das ações da comissão na qualificação do planejamento da alta no hospital universitário.

### Contribuições para as áreas da enfermagem e saúde

Esta pesquisa possibilitou instituir e implantar uma CGAH comprometida e articulada com o serviço. O resultado desta pesquisa contribui para qualificação do serviço de enfermagem e demais profissionais de saúde, visando ao preparo de pacientes e familiares para continuidade do cuidado em domicílio. Ademais, colabora com a alta segura e ágil e com a redução de eventos adversos evitáveis no domicílio, responsáveis por readmissões.

## CONSIDERAÇÕES FINAIS

O estudo instituiu e implantou a CGAH no hospital universitário e avaliou a percepção do grupo sobre o planejamento e implantação da CGAH para o acompanhamento de pessoas com alta dependência e seus cuidadores. Para isso, elaborou instrumentos e fluxos como critérios de elegibilidade, fluxo de captação e admissão dos pacientes, escala de prontidão e fluxos de acompanhamento, de alta e pós-alta, construindo um modelo para apoiar pacientes e cuidadores na continuidade do cuidado.

A percepção do grupo foi positiva tanto para o método utilizado quanto para o tema e a repercussão da comissão em suas práticas. Isso é importante para dar continuidade à CGAH. O envolvimento de profissionais de diferentes categorias ampliou o cenário de percepções das necessidades de intervenção e orientação em saúde para pacientes e familiares. O sentimento de pertencimento ao coletivo foi fortalecido por meio da valorização de cada contribuição individual e pelo empoderamento teórico diante da temática a ser discutida e trabalhada na instituição.

Os fluxos e instrumentos construídos deverão ser validados futuramente para que possam ser reproduzidos em outros espaços de assistência, assim como deverá ocorrer uma avaliação do impacto da CGAH nos pacientes acompanhados.

Este resultado serve de modelo para outras iniciativas, especialmente em hospitais que recebem estudantes. A CGAH do hospital universitário visa seu o desenvolvimento e ampliação para todas as unidades do hospital, além da articulação com o Núcleo Interno se Regulação, implantado após a finalização desta PA.

## Data Availability

Os dados de pesquisa estão disponíveis no corpo do artigo.
